# Exposure to Trihalomethanes through Different Water Uses and Birth Weight, Small for Gestational Age, and Preterm Delivery in Spain

**DOI:** 10.1289/ehp.1002425

**Published:** 2011-08-02

**Authors:** Cristina M. Villanueva, Esther Gracia-Lavedán, Jesús Ibarluzea, Loreto Santa Marina, Ferran Ballester, Sabrina Llop, Adonina Tardón, Mariana F. Fernández, Carmen Freire, Fernando Goñi, Xavier Basagaña, Manolis Kogevinas, Joan O. Grimalt, Jordi Sunyer, on behalf of the INMA (Infancia y Medio Ambiente) Project

**Affiliations:** 1 Centre for Research in Environmental Epidemiology (CREAL), Barcelona, Spain; 2 IMIM (Hospital del Mar Research Institute), Barcelona, Spain; 3 CIBER Epidemiología y Salud Pública (CIBERESP), Spain; 4 Subdirección de Salud Pública, Gipuzkoa, Spain; 5 Centro Superior de Investigación en Salud Pública (CSISP), Valencia, Spain; 6 Universitat de València, València, Spain; 7 Universidad de Oviedo, Oviedo, Spain; 8 Hospital Universitario San Cecilio, Universidad de Granada, Granada, Spain; 9 Laboratorio de Salud Pública, Gobierno Vasco, Spain; 10 National School of Public Health, Athens, Greece; 11 Institute of Environmental Assessment and Water Research (IDAEA), Centro Superior de Investigaciones Científicas (CSIC), Barcelona, Spain

**Keywords:** birth weight, cohort study, disinfection by-products, epidemiology, low birth weight, newborn, premature birth, small for gestational age, trihalomethanes, water pollution

## Abstract

Background: Evidence associating exposure to water disinfection by-products with reduced birth weight and altered duration of gestation remains inconclusive.

Objective: We assessed exposure to trihalomethanes (THMs) during pregnancy through different water uses and evaluated the association with birth weight, small for gestational age (SGA), low birth weight (LBW), and preterm delivery.

Methods: Mother–child cohorts set up in five Spanish areas during the years 2000–2008 contributed data on water ingestion, showering, bathing, and swimming in pools. We ascertained residential THM levels during pregnancy periods through ad hoc sampling campaigns (828 measurements) and regulatory data (264 measurements), which were modeled and combined with personal water use and uptake factors to estimate personal uptake. We defined outcomes following standard definitions and included 2,158 newborns in the analysis.

Results: Median residential THM ranged from 5.9 μg/L (Valencia) to 114.7 μg/L (Sabadell), and speciation differed across areas. We estimated that 89% of residential chloroform and 96% of brominated THM uptakes were from showering/bathing. The estimated change of birth weight for a 10% increase in residential uptake was –0.45 g (95% confidence interval: –1.36, 0.45 g) for chloroform and 0.16 g (–1.38, 1.70 g) for brominated THMs. Overall, THMs were not associated with SGA, LBW, or preterm delivery.

Conclusions: Despite the high THM levels in some areas and the extensive exposure assessment, results suggest that residential THM exposure during pregnancy driven by inhalation and dermal contact routes is not associated with birth weight, SGA, LBW, or preterm delivery in Spain.

Birth weight and gestational age influence health status in childhood and later in life. Low birth weight (LBW) increases the risk of infant mortality and morbidity (McCormick 1985), as well as disease in adulthood, including coronary heart disease, stroke, type 2 diabetes mellitus, adiposity, metabolic syndrome, and osteoporosis (Gluckman et al. 2008), among other outcomes (Strauss 2000). Infant morbidity is inversely related to gestational age, and preterm deliveries may have long-term effects on adult health (Saigal and Doyle 2008). Environmental exposures during pregnancy, such as exposure to water contaminants (Windham and Fenster 2008), may influence birth weight and duration of gestation.

Disinfection by-products (DBPs) are unintended widespread contaminants of drinking water and swimming pools formed during disinfection. Small DBP molecules can pass from the mother to the fetus through the placenta (Danielsson et al. 1986; Dowty et al. 1976). Evidence from animal experiments shows a range of adverse reproductive effects, including sperm toxicity, decreased fertility, delayed puberty, teratogenicity, fetotoxicity, and reduced fetal growth and survival (Tardiff et al. 2006). Some of these effects have been evaluated in observational studies in humans. The body of evidence suggests that measures of reduced fetal growth such as intrauterine growth retardation, small for gestational age (SGA), and LBW are the outcomes most consistently associated with DBP exposure during pregnancy (Tardiff et al. 2006). Epidemiological evidence concerning effects on preterm delivery or reduced gestation length is inconsistent (Hoffman et al. 2008a; Lewis et al. 2007; Wright et al. 2004).

DBPs constitute complex mixtures of > 600 identified chemicals with diverse physicochemical and toxic properties (Richardson et al. 2007). Trihalomethanes (THMs), the DBPs found generally at highest concentrations, are regulated in drinking water in the United States, Canada, the European Union, and other countries. The most common THMs are chloroform, bromodichloromethane, dibromochloromethane, and bromoform. Exposure routes include ingestion and inhalation and dermal absorption during showering, bathing, and swimming in pools, given the volatility and skin permeability of THMs. Experimental studies show that showering, bathing, and dishwashing are the household water uses that lead to the highest concentrations of THMs in blood (Ashley et al. 2005). According to a recent meta-analysis (Grellier et al. 2010), the SGA risk associated with THM exposure during pregnancy is small but statistically significant. However, individual-based studies with detailed exposure assessment are sparse, and evidence concerning dose–response relationships is inconclusive.

We conducted a longitudinal study in five Spanish areas with heterogeneous levels of DBPs to estimate exposure to THMs during pregnancy through ingestion, showering, bathing, and swimming in pools and to evaluate the association of these exposures with birth weight, SGA, LBW, and preterm delivery.

## Materials and Methods

*Study design and population.* A mother–child cohort study was set up in four Spanish areas following a common protocol to constitute the INMA [Infancia y Medio Ambiente (Environment and Childhood)] Project (INMA 2011). Study subjects in Asturias, Gipuzkoa, Sabadell, and Valencia were recruited at the 12th week of gestation and followed until delivery. A previous mother–child hospital-based cohort in Granada also was incorporated into the INMA Project [for a detailed description of the study areas, see Supplemental Material (http://dx.doi.org/10.1289/ehp.1002425)]. Eligibility criteria for enrollment were maternal age ≥ 16 years, singleton pregnancy, planning to deliver at the study hospitals, being able to communicate in either of the official languages, and not having followed an assisted reproduction program (Guxens et al. 2011). The study sample was representative of the target population in terms of prenatal care attendance in the public health system (used by > 80% of the pregnant women); however, the educational level was higher than the target population average. From 45% to 98% of the eligible pregnant women agreed to participate, and enrollment periods ranged from October 2000 in Granada to February 2008 in Gipuzkoa (Guxens et al. 2011). Numbers of recruited subjects at week 12 of gestation were 494 in Asturias, 638 in Gipuzkoa, 657 in Sabadell, and 827 in Valencia. At week 32, 485 women in Asturias, 618 in Gipuzkoa, 628 in Sabadell, and 794 in Valencia were interviewed. There were 668 eligible subjects at gestation in Granada. From the initial sample at recruitment, 485 women (98%) in Asturias, 611 (96%) in Gipuzkoa, 620 (95%) in Sabadell, 787 (95%) in Valencia, and 502 (75%) in Granada were followed until delivery and confirmed informed consent for themselves and their children to participate. The study was approved by the ethical committees of the participating centers, and all subjects gave written consent at enrollment and delivery. Only living newborns were included in the study.

*Water use during pregnancy.* The interview at week 32 of gestation included questions on water use during pregnancy: source of drinking water (municipal, bottled, private well, other) at home and outside the home, use of a home water filter, changes in water ingestion since getting pregnant, and frequency and duration of showering, bathing, and swimming pool attendance (indoors and outdoors during winter and summer). Tap water ingestion was ascertained at weeks 12 and 32 using a food frequency questionnaire that queried intake of tap water and beverages made with tap water (nine categories of 250-mL glass consumption: never or < 1/month; 1–3/month; 1, 2–4, or 5–6/week; 1, 2–3, 4–5, or ≥ 6/day). A continuous variable (liters per day) was computed using the midpoint of each category. Overall, 10% of women reported that they changed the type of water used for drinking or cooking water since getting pregnant (range, 6% in Sabadell to 14% in Asturias). The mean ± SD intake was 0.44 ± 0.6 L/day in week 12 and 0.46 ± 0.67 L/day in week 32. The percentages of women reporting different categories of tap water intake between the two reporting periods ranged from 15% (Sabadell) to 54% (Gipuzkoa). Ingestion of water-based fluids (coffee, herbal drinks, and soup) and source of water for cooking were also obtained but not further used because THM levels in food are modified from levels in tap water (Huang and Batterman 2009). In Granada, water use during pregnancy was collected retrospectively from 132 women in 2008, 6–8 years after delivery, using the same questions as for the rest of the cohort.

*THM levels.* Chlorine was the main disinfectant used for drinking water in all the study areas. Levels of THMs were ascertained based on sampling campaigns and regulatory data from local authorities and water companies. The sampling strategy did not consider individual pregnancy periods but attempted to represent the period between the minimum and maximum conception dates of study subjects, except for the Granada cohort. Measurements were conducted at different time points: 2004–2008 (Asturias), 2006–2008 (Gipuzkoa), 2004–2006 (Granada), 2004–2006 (Sabadell), and 2004–2005 (Valencia). Sampling locations were defined to be geographically representative of the study areas, and water samples were collected from taps with no filtration or other treatments that could affect THM concentration. THMs were determined in 183 samples in Asturias (18 from our own sampling and 165 from regulatory measurements), 421 in Gipuzkoa (own sampling), 128 in Granada (79 own sampling, 49 regulatory), 198 in Sabadell (148 own sampling, 50 regulatory), and 162 in Valencia (own sampling). Water samples in swimming pools were collected from municipalities that accounted for the top 70% or more of the study population within each cohort (13 municipalities). For details on the sampling campaigns and the THM analyses, see Supplemental Material (http://dx.doi.org/10.1289/ehp.1002425).

*THM modeling.* Comparison of mean THM concentrations based on regulatory surveys and our own measurements did not show significant differences (*p*-value from *t*-test > 0.10), and data from both sources were used. Separate models for each area were conducted to predict chloroform, bromodichloromethane, dibromochloromethane, bromoform, and total THM and to assign a concentration to the distribution system of the municipality where women resided. For the modeling procedure and tested variables and for details of the final prediction models, see Supplemental Material, Tables 1 and 2 (http://dx.doi.org/10.1289/ehp.1002425). Final models predicted average monthly THMs levels from conception until delivery in each participant’s residential water supply. Estimates of residential THM level were calculated for 455 subjects in Asturias, 592 in Gipuzkoa, 572 in Sabadell, 727 in Valencia, and 199 in Granada. Estimation of THM levels was not possible for all pregnancies followed to delivery because of missing THM data in some municipalities, missing or incomplete address, or missing gestational age.

**Table 1:** Characteristics of the study population.

Variable	Overall*a* (*n* = 2,074)	Asturias (*n* = 387)	Gipuzkoa (*n* = 512)	Sabadell (*n* = 513)	Valencia (*n* = 662)	Granada (*n* = 84)
Newborn												
Male sex (%)		51.6		50.4		51.4		51.7		52.6		100
Mean birth weight (g)		3,268		3,268		3,322		3,254		3,236		3,415
Mean weeks of gestation		39.7		39.6		39.8		39.7		39.6		39.4
SGA (%)		10.6		9.0		8.6		11.5		12.2		2.4
Preterm (%)		3.7		3.9		2.5		2.7		5.3		11.9
LBW (%)		4.6		5.4		3.3		4.3		5.4		1.2
Mother												
Mean age (years)		30.7		31.7		31.4		30.3		29.9		31.6
Mean height (cm)		162.7		162.3		164.0		162.4		162.0		162.3
Mean prepregnancy weight (kg)		62.4		62.2		61.9		62.5		62.7		62.2
Education (%)*b*												
Primary		24		18		14		27		34		18
Secondary		42		44		35		43		44		44
University		34		38		51		30		22		38
Social class (%)*b*												
I, II		21		21		30		20		15		21
III		26		22		28		30		25		22
IV, V		53		57		42		50		60		57
Living with the father (%)		98		98		99		99		98		98
Nulliparous (%)		57		62		54		58		56		62
Mean weight gain during pregnancy (kg/week)		0.42		0.42		0.42		0.44		0.42		0.42
Smoked during pregnancy (%)		17		18		11		14		23		18
Mean paternal weight (kg)*c*		80.5		82.1		80.3		80.1		80.2		82.1
Season of conception (%)												
Winter		25		27		17		21		31		27
Spring		25		21		30		25		25		21
Summer		26		22		30		30		22		22
Autumn		24		30		23		24		22		30
**a**Includes Asturias, Gipuzkoa, Sabadell, and Valencia. **b**Percentages do not always add to 100 because of missing data. **c**Numbers of missing values are 14 in Asturias, 10 in Gipuzkoa, 7 in Sabadell, 1 in Valencia, 14 in Granada, and 32 overall (excludes Granada).												

**Table 2:** Water use during pregnancy in the study population.

Variable	Overall*a* (*n* = 2,074)	Asturias (*n* = 387)	Gipuzkoa (*n* = 512)	Sabadell (*n* = 513)	Valencia (*n* = 662)	Granada (*n* = 84)
Source of drinking water at home (%)												
Bottle		61.2		51.9		24.6		88.3		73.9		21.7
Tap filtered		6.3		4.7		9.6		5.7		5.1		8.4
Tap nonfiltered		27.1		35.7		64.1		2.9		12.1		65.1
Other		5.4		7.7		1.6		3.1		8.9		4.8
Missing (*n*)												1
Tap water (among those drinking unfiltered tap water) (L/day)												
50th (25th–75th) percentile		1.2 (0.9–1.4)		1.2 (0.6–1.8)		1.1 (0.9–1.4)		1.4 (0.9–1.8)		1.3 (0.9–1.8)		1.1 (0.6–1.1)
*n*		561		138		328		15		80		54
Showering/bathing (%)												
Shower only		88		91		93		86		85		84
Bath only		2		3		2		2		2		5
Shower and bath		10		6		5		12		13		11
Shower frequency (times/week)												
50th (25th–75th) percentile		7 (6–7)		7 (7–7)		7 (5–7)		7 (6–7)		7 (5–7)		7 (6–7)
*n*		2,034		375		503		504		652		80
Shower duration (min)												
50th (25th–75th) percentile		10 (7–15)		10 (6–15)		10 (5–15)		10 (7–15)		10 (10–15)		10 (5–10)
*n*		2,033		375		503		504		651		80
Bath frequency (times/month)												
50th (25th–75th) percentile		2 (1–3)		2 (1–7)		2 (1–3)		1 (1–2)		2 (1–3)		2 (1–3)
*n*		242		34		37		74		97		13
Bath duration (min)												
50th (25th–75th) percentile		30 (20–30)		30 (15–35)		20 (15–30)		30 (20–30)		30 (15–30)		20 (15–20)
*n*		242		34		37		73		98		13
Swimming in pools (%)												
Yes		43		26		28		51		58		49
No		57		74		72		49		42		51
Missing												1
Outdoor pools (min/month)												
50th (25th–75th) percentile		35 (15–90)		38 (20–120)		30 (13–75)		30 (15–113)		34 (15–90)		53 (30–150)
*n*		641		41		71		193		336		38
Indoor pools (min/month)												
50th (25th–75th) percentile		85 (30–150)		113 (45 –225)		70 (30–120)		40 (15–90)		135 (60–270)		0 (0, 0)
*n*		328		55		82		108		83		41
**a**Includes Asturias, Gipuzkoa, Sabadell, and Valencia.												

*Exposure indices.* The modeled residential THM level was multiplied by daily personal water use and uptake factors from the literature, to derive an estimate of daily THMs concentration in the bloodstream (Whitaker et al. 2003), as described in Supplemental Material, Table 3 (http://dx.doi.org/10.1289/ehp.1002425). Chloroform and brominated THM were analyzed separately because toxic properties differ among species, particularly brominated versus chlorinated species. A 90% reduction in ingestion was applied if a home filter was used (Egorov et al. 2003; Weinberg et al. 2006). We averaged the 12- and 32-week tap water intakes to compute the ingested THM. Average THM uptake in the first, second, and third trimester and the whole pregnancy were calculated. Bathing and showering uptakes were added, and total household uptake was calculated by adding ingestion, showering, and bathing. Because of missing data in water use variables, residential THM uptake were calculated for 2,386 subjects (425 in Asturias, 576 in Gipuzkoa, 560 in Sabadell, 720 in Valencia, and 105 in Granada). To estimate swimming pool uptake, study area–specific THM averages were calculated for indoor and outdoor pools. Second, personal attendance at indoor and outdoor pools was multiplied by the area-THM average. To obtain an overall swimming pool attendance index, indoor and outdoor uptakes were added.

**Table 3:** Estimated change in birth weight (g) expressed as β-coefficients (95% CIs) from a linear regression*a* for a 10% increase in THM uptake (μg/day) in the second trimester.

Exposure	Overall*b*	Asturias	Gipuzkoa	Sabadell	Valencia	Granada
Ingestion at the residence												
Chloroform		–0.44 (–1.01, 0.13)		–1.09 (–2.58, 0.40)		–0.45 (–1.67, 0.78)		0.59 (–1.30, 2.48)		–0.60 (–1.39, 0.19)		–0.31 (–3.09, 2.47)
Brominated THM		–0.41 (–1.05, 0.22)		–1.14 (–2.64, 0.36)		–0.38 (–1.79, 1.02)		0.53 (–1.24, 2.30)		–0.62 (–1.56, 0.31)		0.28 (–2.38, 2.93)
*n*		2,074		387		502		505		662		72
Showering/bathing												
Chloroform		–0.39 (–1.30, 0.51)		–1.19 (–5.18, 2.81)		–1.85 (–5.24, 1.54)		1.34 (–2.70, 5.37)		–0.25 (–1.31, 0.80)		3.30 (–1.91, 8.52)
Brominated THM		0.20 (–1.32, 1.72)		0.05 (–5.32, 5.43)		–2.00 (–6.64, 2.64)		–0.18 (–5.36, 4.99)		0.85 (–1.03, 2.74)		4.51 (–0.47, 9.48)
*n*		2,074		387		502		505		662		72
Total residential												
Chloroform		–0.45 (–1.36, 0.45)		–1.99 (–6.03, 2.04)		–2.26 (–5.86, 1.33)		1.33 (–2.78, 5.44)		–0.28 (–1.34, 0.78)		2.89 (–2.34, 8.12)
Brominated THM		0.16 (–1.38, 1.70)		–0.41 (–5.96, 5.14)		–2.38 (–7.31, 2.55)		–0.22 (–5.46, 5.02)		0.84 (–1.05, 2.73)		4.39 (–0.60, 9.38)
*n*		2,074		387		502		505		662		72
Swimming pool												
Chloroform		–0.10 (–0.68, 0.47)		1.65 (0.08, 3.23)*		–1.31 (–2.75, 0.13)		0.15 (–0.76, 1.05)		–0.26 (–1.39, 0.87)		–0.05 (–5.69, 5.60)
Brominated THM		–0.10 (–0.68, 0.48)		1.65 (0.08, 3.23)*		–1.31 (–2.75, 0.13)		0.15 (–0.78, 1.08)		–0.25 (–1.37, 0.86)		–0.05 (–5.69, 5.60)
*n*		2,054		379		500		503		654		68
**a**Overall results are adjusted for weeks of gestation (linear and quadratic); sex; parity; maternal height and weight, weight gain, and smoking during pregnancy; and cohort. Area-specific results are adjusted for these variables in addition to maternal education and season of conception (Asturias and Granada); social class, marital status, and paternal weight (Gipuzkoa); education, paternal weight, and season of conception (Sabadell); and social class (Valencia). **b**Excluding Granada. *p < 0.05.												

*Outcomes.* Birth weight was recorded by trained midwives at delivery. Gestational age was calculated from the date of the last menstrual period (LMP) reported at recruitment and was confirmed using ultrasound examination in week 12 of gestation. If gestational age based on reported LMP and ultrasound differed by ≥ 7 days (12% of newborns), duration of gestation was recalculated using a formula based on crown–rump length from an early ultrasound measurement (Westerway et al. 2000). Final gestational age ranged between 23.4 and 42.3 weeks. Birth weights < 10th percentile for gestational age and sex according to national growth curves (Carrascosa et al. 2004) were defined as small for gestational age (SGA). Deliveries before 37 weeks of gestation were defined as preterm births. Birth weight < 2,500 g was defined as LBW (World Health Organization 1995).

*Covariates.* Variables potentially influencing the birth outcomes based on previous knowledge were considered. Maternal age, height, prepregnancy weight, education, marital status, parity, and country of origin and paternal weight were collected at the week 12 interview. Smoking during pregnancy was recorded at the week 32 interview. Maternal weight gain during pregnancy was computed as the rate of weight gain during the second and third trimester in kilograms per week (Rasmussen and Yaktine 2009), adjusted for gestational age at the last available weight measure to correct for possible heteroskedasticity and nonlinearity of the rate (Dietz et al. 2006). Maternal social class was coded from the longest-held job during the pregnancy, using the four-digit Spanish classification (Instituto Nacional de Estadística 1994), which is closely related to the International Standard Classification of Occupations (ISCO 88): Those in social class I are managers of companies with ≥ 10 employees, senior technical staff, higher-level professionals; II, managers of companies with < 10 employees, intermediate-level professionals; III, administrative and financial management supporting personnel, other self-employed professionals, supervisors of manual workers, other skilled nonmanual workers; IV, skilled and partly skilled manual workers; V, unskilled manual workers. LMP date was used as conception date.

*Statistical analysis.* Chloroform and brominated THM uptakes were log transformed to normalize the distribution. Because logarithm of zero values in tap water ingestion and swimming pool attendance from bottled water consumers or nonswimmers led to invalid transformed variables, these were imputed arbitrarily half the area-specific lowest value for ingestion and swimming, respectively. We evaluated the association between birth weight and log THM uptake by linear regression adjusting for gestational age and other potential confounders. Fractional polynomials were applied to identify the best transformation of gestational age in the birth weight regression models, because fetal weight gain is not constant over pregnancy. Statistically significant covariates (*p*-value < 0.05) and variables resulting in a ≥ 10% change in the ®-coefficient for log THM were retained in the models. Logistic regressions were used to estimate odds ratios (ORs) for dichotomous outcomes adjusting for potential confounders, and generalized additive models were used to evaluate the shape of the dose–response curve.

Coefficients from the regression models were multiplied by the logarithm of 1.1 to derive an effect estimate for a 10% increase in exposure. Analyses were stratified by region, and comparable cohorts in terms of design and water data collection (Asturias, Gipuzkoa, Sabadell, and Valencia) were combined. Previous evidence suggests that the vulnerable window for exposure could be the second trimester (Hoffman et al. 2008a; Lewis et al. 2006; Porter et al. 2005) or third trimester (Hinckley et al. 2005; Wright et al. 2004). We used exposure in the second trimester in the main models to maximize the sample size, because some pregnancies did not reach the third trimester. We estimated the effect of exposure during the first and third trimesters and overall exposure during pregnancy in alternative models.

In sensitivity analyses, we weighted models for the Valencia and Granada cohorts based on the geographical variability, following the method suggested by Waller et al. (2001). Point estimates and confidence intervals (CIs) did not change substantially (results not shown). A meta-analysis was conducted to compare pooled estimates for the individual study areas with the overall analyses adjusted for cohort. A sensitivity analysis excluding observations with residuals > 3 SDs and < 3 SDs showed no differences, so all observations were included. Subjects who changed residence between weeks 12 and 32 of gestation (5% overall, 3% in Asturias, 3% in Gipuzkoa, 5% in Sabadell, 7% in Valencia) were excluded from the analyses to minimize exposure misclassification. Subjects excluded for changing residence and for missing values in covariates led to final models with smaller numbers compared with the numbers of exposure estimates.

## Results

**Figure 1:**
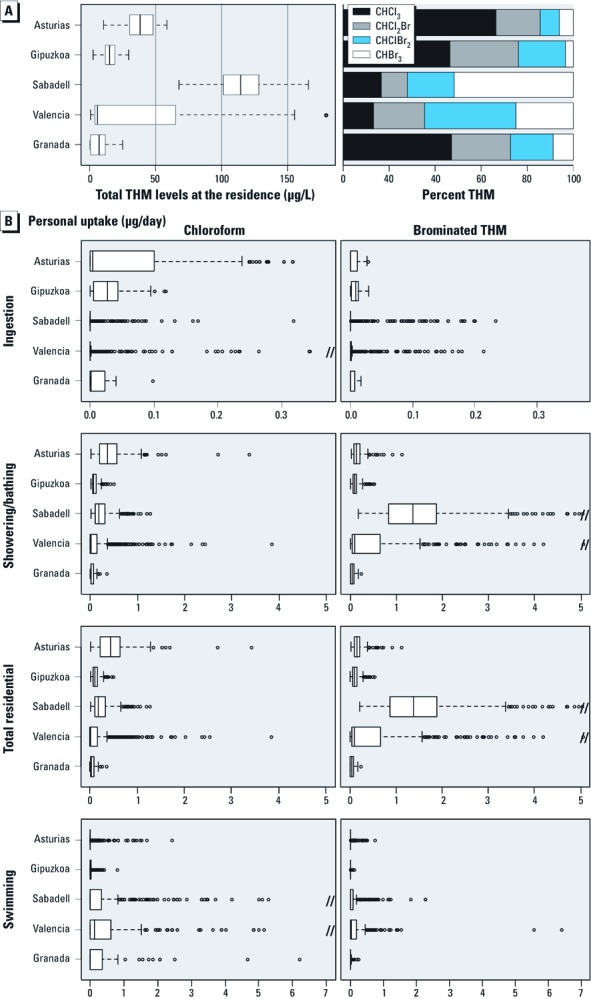
Estimated individual THM levels (μg/L) at the residence (*A*), and uptake (*B*) through different uses in the second trimester and through swimming pool uptake in the whole pregnancy (μg/day). Boxes represent 25th and 75th percentiles; the central line represents the median. Whiskers (dashed lines) indicate ± 1.5 IQR; points outside the whiskers are outliers.

Table 1 describes the characteristics of the study population. Except Granada, which included only male births, the prevalence of most outcomes was similar among the cohorts, and differences were not statistically significant, except for preterm delivery. Differences in maternal age, height, education, social class, weight gain, and smoking during pregnancy and season of conception appeared among cohorts. Table 2 shows the habits of residential water ingestion and swimming pool attendance. Most women consumed bottled water outside the home (95% in Sabadell, 92% in Valencia, 88% in Asturias, 79% in Gipuzkoa, 36% in Granada, and 87% overall); the overall bottled water consumption excluding Granada is 89%. Overall, 43% of women attended swimming pools during pregnancy, ranging from 26% (Asturias) to 58% (Valencia). Levels of THMs in swimming pools are shown in the Supplemental Material, Figure 1 (http://dx.doi.org/10.1289/ehp.1002425). Residential ingestion uptake was very low, and most of the total residential uptake resulted from showering/bathing (Figure 1). Ingestion contributed 11% to the total residential chloroform uptake and 4% of brominated THM uptake.

Final sample sizes differed slightly among outcomes because of missing outcome and covariate data. Because models for combined analyses of data from Asturias, Gipuzkoa, Sabadell, and Valencia had fewer covariates (fewer missing data) than did area-specific models, we based combined estimates on larger numbers of observations. Crude and adjusted models gave very similar results, so we report only the latter here. Table 3 shows the estimated changes in birth weight associated with a 10% increase in total residential THM uptake. ®-Coefficients were close to the null or not statistically significant for ingestion, showering/bathing, and total residential uptakes. Overall, birth weight was reduced 0.45 g (95% CI: –1.36, 0.45) for total residential chloroform uptake and increased 0.16 g (–1.38, 1.70) for total brominated THM uptake. The meta-analysis gave combined coefficients of –0.42 g (–1.38, 0.54) for chloroform and 0.31 g (–1.29, 1.90) for brominated THMs. The analysis for quartiles of THM uptake showed a lack of association in all categories (data not shown). Swimming pool uptake was associated with an increased birth weight of 1.65 g (0.08, 3.23) in Asturias and a nonsignificant association in the other cohorts, with an overall coefficient of –0.1 g (–0.7, 0.5) for chloroform and bromoform uptake.

Table 4 shows the ORs for preterm delivery. Effect estimates for individual study areas and for the four areas combined (excluding Granada) were similar, as were meta-analysis estimates for uptake of chloroform [1.004 (95% CI: 0.988, 1.019)] and brominated THM [1.005 (0.979, 1.032)] in the residence.

**Table 4:** ORs (95% CIs)*a* of preterm delivery for a 10% increase in THM uptake (μg/day) in the second trimester.

Exposure	Overall*b*	Asturias	Gipuzkoa	Sabadell	Valencia	Granada
Ingestion at the residence												
Chloroform		1.004 (0.995, 1.013)		1.001 (0.972, 1.032)		1.001 (0.975, 1.028)		1.006 (0.965, 1.048)		1.006 (0.995, 1.017)		1.008 (0.975, 1.042)
Brominated THM		1.006 (0.996, 1.016)		0.999 (0.970, 1.030)		1.007 (0.974, 1.040)		1.005 (0.970, 1.042)		1.008 (0.995, 1.021)		1.004 (0.975, 1.034)
*n*		2,071		387		511		506		662		72
Showering/bathing												
Chloroform		0.992 (0.979, 1.006)		0.960 (0.890, 1.034)		0.972 (0.908, 1.040)		1.078 (0.966, 1.202)		1.005 (0.989, 1.022)		1.074 (0.973, 1.182)
Brominated THM		0.999 (0.975, 1.023)		0.923 (0.827, 1.029)		1.003 (0.902, 1.101)		1.089 (0.957, 1.240)		1.008 (0.979, 1.037)		1.035 (0.960, 1.117)
*n*		2,071		387		511		506		662		72
Total residential												
Chloroform		0.993 (0.980, 1.007)		0.968 (0.900, 1.040)		0.969 (0.905, 1.039)		1.090 (0.973, 1.220)		1.006 (0.990, 1.023)		1.065 (0.969, 1.171)
Brominated THM		1.000 (0.976, 1.024)		0.925 (0.828, 1.033)		1.001 (0.897, 1.119)		1.093 (0.960, 1.246)		1.009 (0.980, 1.038)		1.033 (0.958, 1.113)
*n*		2,071		387		511		506		662		72
Swimming pool												
Chloroform		0.989 (0.978, 0.999)*		0.999 (0.963, 1.036)		0.996 (0.966, 1.027)		0.981 (0.956, 1.007)		1.006 (0.987, 1.026)		0.999 (0.927, 1.076)
Brominated THM		0.989 (0.979, 1.000)		0.999 (0.963, 1.036)		0.996 (0.966, 1.027)		0.980 (0.955, 1.007)		1.008 0.989, 1.028)		0.999 (0.927, 1.076)
*n*		2,051		379		509		504		654		68
**a**Overall results are adjusted for birth weight; sex; parity; maternal height and weight, weight gain, and smoking during pregnancy; and cohort. Area-specific results are adjusted for these variables in addition to maternal education (Asturias); maternal education and season of conception (Gipuzkoa); maternal social class, paternal weight, and season (Sabadell); maternal education and season of conception (Valencia); and maternal education (Granada). **b**Excluding Granada. *p < 0.05.												

Table 5 shows the associations for LBW, SGA, birth weight, and preterm delivery by exposure trimester and overall. Associations differed minimally among trimesters in independent models. Models adjusting simultaneously for all trimesters led to minimal differences in birth weight change, LBW, and SGA risks. Risk of preterm delivery and brominated THM uptake differed between exposure in the first and third trimesters, although associations were small (OR, 0.90–1.15). ORs of LBW without adjusting for gestational age were very similar to adjusted ORs.

**Table 5:** OR (95% CI) for change of birth weight (g), SGA, preterm delivery, and LBW for a 10% increase in total residential uptake: overall for Asturias, Gipuzkoa. Sabadell, and Valencia.

Birth weight			LBW			SGA			Preterm delivery		
Exposure	β-Coefficient (95% CI)*a*	*n*	OR (95% CI)*a*	*n*	OR (95% CI)*b*	*n*	OR (95% CI)*c*	*n*
Chloroform																
First trimester		–0.36 (–1.26, 0.55)		2,074		1.000 (0.986, 1.014)		2,074		1.001 (0.993, 1.009)		2,073		0.993 (0.979, 1.007)		2,071
Second trimester		–0.45 (–1.36, 0.45)		2,074		1.001 (0.988, 1.016)		2,074		1.002 (0.994, 1.010)		2,073		0.993 (0.980, 1.007)		2,071
Third trimester		–0.07 (–1.00, 0.85)		2,073		1.004 (0.990, 1.018)		2,073		1.003 (0.995, 1.011)		2,072		1.000 (0.985, 1.014)		2,070
All pregnancy		–0.35 (–1.31, 0.60)		2,074		1.002 (0.988, 1.017)		2,074		1.003 (0.994, 1.011)		2,073		0.994 (0.979, 1.009)		2,071
Mutually adjusted trimesters (one model)				2,073				2,073				2,072				2,070
First trimester		0.12 (–2.07, 2.32)				0.985 (0.943, 1.029)				0.996 (0.978, 1.016)				0.995 (0.960, 1.032)		
Second trimester		–2.40 (–5.22, 0.43)				0.994 (0.939, 1.053)				1.003 (0.979, 1.027)				0.969 (0.927, 1.014)		
Third trimester		2.03 (–0.13, 4.19)				1.023 (0.981, 1.067)				1.003 (0.984, 1.022)				1.035 (1.000, 1.071)		
Brominated THM																
First trimester		–0.24 (–1.78, 1.31)		2,074		1.008 (0.985, 1.032)		2,074		1.003 (0.990, 1.017)		2,073		0.993 (0.970, 1.017)		2,071
Second trimester		0.16 (–1.38, 1.70)		2,074		1.008 (0.986, 1.032)		2,074		1.003 (0.990, 1.016)		2,073		1.000 (0.976, 1.024)		2,071
Third trimester		0.36 (–1.19, 1.92)		2,073		1.007 (0.984, 1.031)		2,073		1.003 (0.990, 1.017)		2,072		1.006 (0.982, 1.031)		2,070
All pregnancy		0.10 (–1.46, 1.66)		2,074		1.008 (0.985, 1.032)		2,074		1.003 (0.990, 1.017)		2,073		1.000 (0.976, 1.024)		2,071
Mutually adjusted trimesters				2,073				2,073				2,072				2,070
First trimester		–5.92 (–11.89, 0.05)				1.002 (0.907, 1.106)				1.004 (0.952, 1.059)				0.902 (0.817, 0.996)*		
Second trimester		1.85 (–6.29, 9.99)				1.031 (0.891, 1.192)				0.994 (0.925, 1.068)				0.996 (0.865, 1.146)		
Third trimester		4.12 (–1.85, 10.08)				0.975 (0.878, 1.084)				1.005 (0.954, 1.058)				1.115 (1.007, 1.235)*		
**a**Adjusted for sex, weeks of gestation (linear and quadratic); parity; maternal height and weight, weight gain, and smoking during pregnancy; and cohort. **b**Adjusted for parity; maternal height and weight, weight gain, and smoking during pregnancy; and cohort. **c**Adjusted for sex; birth weight; parity; maternal height and weight, weight gain, and smoking during pregnancy; and cohort. *p < 0.05.																

Results were similar when we excluded LBW, very low birth weight (< 1,500 g), or preterm deliveries, when we stratified estimates by social class (I, II, III vs. IV, V) or smoking status during pregnancy, and when we adjusted estimates for social class (data not shown). Results from a generalized additive model confirmed the assumption of linearity between the exposure and evaluated effects (data not shown).

## Discussion

Residential exposure to THMs during pregnancy was not associated with birth weight, SGA, LBW, or preterm birth, despite high levels of THMs in some areas. Results were consistent between areas. A high proportion of women consumed bottled water. Showering contributed the highest fraction to our calculated THM exposure in the residence, indicating that inhalation and dermal contact were the main exposure routes in this study population.

The literature concerning birth weight, SGA, LBW, and THM exposure during pregnancy is extensive, but differences in the characteristics of study populations, exposures, and methodologies (including exposure assessment) hamper comparisons among studies. The body of evidence suggests that reduced fetal growth (e.g., SGA, LBW) is a likely effect of exposure (Tardiff et al. 2006), but evidence is far from conclusive and results of a recent meta-analysis of SGA that included studies with a wide range of exposure levels suggests that the actual risk may be small (Grellier et al. 2010). We did not identify evidence of increased risks, consistent with several other studies, including a recent study with detailed assessment of exposure to THMs and haloacetic acids based on personal uptake calculations comparable to ours (Hoffman et al. 2008b). We cannot rule out nondifferential exposure misclassification due to areawide exposure estimates, which may be partly responsible for null findings. Low exposure levels are not a likely explanation for our null findings, given the consistent results among areas with very different levels, including one area (Sabadell) with THM levels that are among the highest described in the literature (Grellier et al. 2010). A speculative explanation for our negative results compared with previous positive findings could be uncontrolled confounding or effect modification by diet or other lifestyle factors, particularly if our populations were of higher socioeconomic status than other study populations. Because oxidative stress is a potential mechanism of action of DBPs (Beddowes et al. 2003), the Mediterranean diet, which is rich in antioxidants, might protect our population relative to others. Compared with the limited previous studies that included personal water use, our study population had low exposure through ingestion, which involves a different mixture of THMs and different internal distribution and metabolic pathways compared with inhalation and dermal exposure routes (Ashley et al. 2005; Leavens et al. 2007). Our results suggest that chemicals incorporated through inhalation and dermal contact do not pose a significant risk of reduced birth weight or preterm delivery. However, these results cannot be extrapolated to the ingested mixture.

Evidence of an association between preterm delivery and THM exposure during pregnancy is mixed, with most of the studies finding either null associations (Aggazzotti et al. 2004; Jaakkola et al. 2001) or evidence of a protective effect (Hoffman et al. 2008a; Wright et al. 2004). A recent meta-analysis that included nine studies reported a null association for THM and chloroform exposure during different pregnancy time windows (Grellier et al. 2010). Our results suggest slight differences by species, exposure windows, and water uses. These inconsistent findings could be attributed partly to chance, although true differences among chemicals and timing of exposure cannot be ruled out for preterm delivery.

Mechanisms of DBP effects on fetal development are not well elucidated. THMs and other DBPs induce oxidative stress, resulting in a depletion of glutathione, increased levels of oxidized DNA and lipid peroxidation (Ahmed et al. 1999; Beddowes et al. 2003), and epigenetic changes through hypomethylation and modified gene expression (Pereira et al. 2001). Because dramatic and concerted changes in methylation and redox (reduction–oxidation) status occur during gestation (Hitchler and Domann 2007), the eventual disruption of the methylation and redox conditions *in utero* could affect the development of the fetus (Gluckman et al. 2008). Effect modification by genetic variants has not been evaluated in epidemiological studies on fetal growth or preterm delivery associated with DBP exposure. Assessment of gene–environment interactions could provide additional evidence on mechanisms and would be an important improvement in future studies.

The inclusion of areas with different total and specific THM levels was a strength of our study, and the large number of potential confounders or effect modifiers collected reduced the probability of spurious or confounded risk estimates. We carefully defined outcome variables following common definitions in all areas, and misclassification is likely to be low or absent. Results were highly consistent among study areas, particularly for residential THM uptake, providing robust combined estimates. The exposure assessment was extensive, including detailed personal information on water habits, THM data and modeling, and the exclusion of subjects who changed residence during pregnancy as a criterion of quality of the exposure estimate. We examined the effect on risk estimates of using exposure metrics weighted for the variability of THM levels within geographical units and found no substantial changes. Previous studies paid little attention to the type of water ingested inside and outside the residence separately, and the differences we observed suggest the relevance of this information. Although the sample size by region was limited in detecting some effects, the overall study population was among the largest in the literature and was sufficient to detect, with statistical significance, even subtle effects on a continuous variable like birth weight.

We based our exposure assessment on THMs, which are the most prevalent group of DBPs. However, THMs are poor surrogates of specific DBPs, particularly DBPs that are nonvolatile and non-skin-permeable. Unfortunately, data on haloacetic acids were available in only one of the cohorts (Santa Marina et al. 2010). Given that THMs may not correlate with DBPs of most toxicological importance, assessment of exposure to other specific DBPs deserves attention in future studies. We based assessment of exposure in swimming pools on a reduced number of THM measurements from selected pools, a few years after the pregnancies. Consequently, exposure misclassification is likely to be higher than for residential THM estimates. This, together with uncontrolled confounders (e.g., physical activity) or biases could partly explain the inconsistency of the risk estimates for swimming pools among areas, particularly for birth weight. The prevalence of LBW and preterm delivery in our study population was low compared with average values in Spain, where 7.6–12.3% of newborns have LBW and 7.4–11.1% are preterm (Figueras et al. 2008). This was probably due to the higher socioeconomic status of our study populations compared with the overall population of Spain (Fernandez-de-Las-Penas et al. 2010). Because socioeconomic status was not a main confounder, this selected population likely has little impact on external validity. Given that we collected data retrospectively from the women in the Granada cohort, the potential for recall bias cannot be ruled out. The potential for recall bias was eliminated because of the prospective nature of the data collection for the remaining cohorts. Because previous studies report changes in water use behaviors during pregnancy (Forssén et al 2009), a potential study limitation was that we collected data on showering, bathing, and swimming habits at only one time point during pregnancy. However, the stable water ingestion habits in our study population suggest that exposure misclassification due to substantial differences in water use during pregnancy was unlikely.

In conclusion, despite the high THM levels found in some study areas and the extensive exposure assessment, our results suggest that residential THM exposure during pregnancy was driven by inhalation and dermal contact routes and was not associated with birth weight (SGA, LBW) or preterm delivery in Spain.

## Supplemental Material

(668 KB) PDF
