# Supplementary material for: Exposure to Trihalomethanes through Different Water Uses and Birth Weight, Small for Gestational Age, and Preterm Delivery in Spain
Source: Environ Health Perspect. 2011 Aug 2;119(12):1824–30. doi: 10.1289/ehp.1002425 (PMC3261969; doi:10.1289/ehp.1002425)
Supplement: (668 KB) PDF [file ehp.1002425.s001.pdf]

SUPPLEMENTAL MATERIAL

Exposure to Trihalomethanes through Different Water Uses and Birth Weight, Small for Gestational Age and Preterm Delivery in Spain

Cristina M Villanueva, Esther Gracia-Lavedán, Jesús Ibarluzea, Loreto Santa Marina, Ferran Ballester, Sabrina Llop, Adonina Tardón, Mariana F. Fernández, Carmen Freire, Fernando Goñi, Xavier Basagaña, Manolis Kogevinas, Joan O Grimalt, Jordi Sunyer

Table of contents

Study area 2

Swimming pool sampling campaigns 2

Experimental trihalomethane analysis in tap and swimming pool water 3

Procedure to model trihalomethane levels during pregnancy 3

Supplemental Material, Table 1. Variables tested in the models to predict residential trihalomethane levels 5

Supplemental Material, Table 2. Final models used to predict average monthly trihalomethane levels in the study population 6

Supplemental Material, Table 3. Uptake factors from the literature and values used in this study 7

Supplemental Material, Figure 1. Boxplots of the total trihalomethane levels in indoor and outdoor swimming pools sampled in the study areas. 8

Supplemental Material, Figure 2. Total trihalomethane levels in the study areas. Average levels are presented for each district in Sabadell, and each municipality in Asturias, Gipuzkoa, Valencia and Granada. The grey circles represent the average level and the black circles inside represent the standard error. The white circle at the top right indicates the scale of concentrations in each area. The number of samples (N) in each cohort are indicated 9

References 10

Study area

The study area in the Asturias cohort comprises 9 municipalities (Avilés, Castrillón, Corvera, Gozón, Muros de Nalón, Pravia, Cudillero, Soto del Barco and Illas) accounting for approximately 160.000 inhabitants in 2010, supplied by surface water through 11 distribution networks. The Gipuzkoa cohort comprises 2 counties (Goierri, Alto Urola) with 25 municipalities and 90.000 inhabitants, supplied by surface water from 3 reservoirs through 26 distribution networks. The Sabadell cohort is comprised by a single municipality with approximately 200.000 inhabitants and a single water distribution network from a surface source. The Valencia cohort includes 34 municipalities and part of Valencia City, accounting for approximately 300.000 inhabitants supplied with water from different sources (surface, ground and mixed) and 70 distribution networks. The Granada cohort includes 40 municipalities and part of Granada city, representing 475.000 inhabitants supplied by ground and surface water through 33 distribution networks. Chlorine is the main disinfectant used in all the study areas during the study period.

Swimming pool sampling campaigns

Public facilities with swimming pools open to the public were identified in the study areas, including sports facilities with indoor and/or outdoor pools and outdoor leisure swimming pools. One of the sampling campaigns was conducted in summer (July 2009) in order to sample simultaneously indoor and outdoor pools for comparison, since outdoor pools are only open in the summer season (mid June to mid September). Two criteria were followed to select swimming pools: municipality-owned swimming pools (since these tend to be the most frequented pools in Spain), and/or facilities with both indoor and outdoor pools. In Asturias, 3 sports complexes with swimming pools open to the public were identified in the study municipalities, 2 in Avilés and 1 in Castrillón. All of them were indoor pools sampled for the study in July 2009. A sample in the adult and infant pool was collected in the 2 swimming pools in Avilés, resulting in a total of 5 samples. Sabadell had 9 indoor swimming pools open to the public (7 of them had also an outdoor pool) and 6 outdoor swimming pools. The 9 sport facilities with indoor swimming pools were contacted and water samples were collected in July 2006 (N=8, indoors), February 2007 (N=8, indoors), July 2007 (N=7, indoors), and July 2009 (N=5 outdoors, N=4 indoors). In Valencia, 17 sports facilities with swimming pools were identified (4 in Paterna, 2 in Burjassot, 2 in Llíria, 1 in Riba-Roja de Turia, 1 in Godella, 1 in Bétera, and 6 in the study area located in Valencia city). The municipal pools of each municipality were contacted and sampled in July 2009, resulting in 8 samples (5 indoors, 3

outdoors), i.e. 1 in Paterna (indoors), 1 in Burjassot (indoors), 2 in Lliria (indoor and outdoors), 1 in Riba-Roja de Turia (outdoor), 1 in Godella (indoor), 1 in Bétera (indoors) and 1 in Valencia (outdoors). In Granada, 9 swimming pools open to the public were identified in the study municipalities (4 in Granada, and 1 each in Armilla, Churriana, Maracena, Alhedin and Albolote). In total, 5 pools were sampled in July 2009, 3 indoors (2 in Granada, one in Armilla) and 2 outdoors (Granada, Churriana). In Gipuzkoa, 20 indoor pools were sampled from January to April 2006, selected as the most frequented among a total of 35 in the province (Santa Marina et al. 2009). All sampled swimming pools used chlorine as disinfectant. The sampled indoor swimming pools represented 100% of indoor pools in Asturias, Sabadell and Granada, 57% in Gipuzkoa and 42% in Valencia study areas. The coverage of outdoor pools was 0% in the Northern cohorts (Asturias and Gipuzkoa), 33% in Granada, 38% in Sabadell and 100% in Valencia. The distribution of THM levels in the sampled swimming pools in Asturias, Granada, Sabadell and Valencia are shown in the Supplemental Material, Figure 1. More details can be found in Font-Ribera et al. 2010. THM levels in Gipuzkoa were previously published (Santa Marina et al. 2009).

Experimental trihalomethane analysis in tap and swimming pool water

Samples were collected in two 40 ml glass vials with 5 mg of sodium thiosulphate, avoiding bubble formation and stored at 4ºC. The vials were sealed with Teflon-faced rubber septa and open-top screw plugs. Chloroform, bromodichloromethane, dibromochloromethane, and bromoform were measured using purge-and-trap gas chromatography/mass spectrometry (GC/MS) (Tekmar 3100, Voyager MS; ThermoFisher, Waltham, MA, USA). Supplemental Material, Figure 2 illustrates the average levels and variability of trihalomethanes (THMs) at the residence.

Procedure to model trihalomethane levels during pregnancy

In a first stage, bivariate descriptive analyses were conducted to identify the variables influencing THM levels (see variables in Supplemental Material, Table 1). Secondly, the THM variable was transformed when necessary to obtain a normal distribution and bivariate linear regressions were conducted. Variables associated with THM levels with statistical significance (p-value<0.05) or variables *a priori* relevant to THMs were included simultaneously in linear regression models and generalized additive models (GAMs) with different combinations of variables. GAM models were used to fit a smoothed function of month that was used to predict levels for months without observations and also a smooth

function of coordinates. Both the R-squared and the Akaike information criteria were used to select the final model (see Supplemental Material, Table 2). The models with highest R2 and lowest AIC were the selected ones. We first modelled total THMs following this procedure. For chloroform, bromodichloromethane, dibromochloromethane and bromoform models the same procedure was followed, with the difference that we started with the variables included in the total THM model. Non-significant variables were removed and relevant variables for temporal or geographical variability, not initially included, were tested and retained if appeared to be statistically significant.

Supplemental Material, Table 1. Variables tested in the models to predict residential trihalomethane levels

Cohort	Variables	
Asturias	Temporal	sampling month<br>sampling year
	Geographical	municipality (9 categories)
	Other	water source (2 categories: surface, mixed)<br>sampling regimen (2 categories: own sampling, regulatory data)
Gipuzkoa	Temporal	sampling month<br>sampling year
	Geographical	sample site (34 categories) [a]
	Other	water source (2 categories: reservoir, spring)
Granada	Temporal	sampling month<br>sampling year
	Geographical	municipality (40 categories)
	Other	water source (2 categories: ground, surface)<br>sampling regimen (2 categories: own sampling, regulatory data)
Sabadell	Temporal	sampling month<br>sampling year
	Geographical	universal transverse mercator (UTM) coordinates[b]<br>district (8 categories)<br>district grouped (3 categories. For total THM and CHBr3 models)<br>district grouped2 (2 categories. For CHCl3 model)<br>district grouped3 (2 categories. For CHClBr2 model)
	Other	sampling regimen (2 categories: own sampling, regulatory data)
Valencia	Temporal	sampling month<br>sampling year
	Geographical	urbanicity (4 categories: urban, metropolitan, semi-rural, rural)<br>population center (49 categories) [c]
	Other	water source (3 categories: ground, surface, mixed)

a. The 34 sampling sites were chosen to represent all municipalities (N=25) and distribution networks (N=26) in the Gipuzkoa cohort. Two sampling points were identified in municipalities with >5000 inhabitants (8 municipalities) and one sampling point in the rest.

b. Coordinates of the actual sampling points were estimated by geocoding the sampling address both from our own and regulatory measurements

c. Population center in Valencia is the result of grouping the distribution systems (N=70) in categories with homogeneous water quality and urbanicity. All the study municipalities (N=34) are represented in the 49 population centers. Measurements of THMs were available in all the distribution systems and municipalities.

Supplemental Material,Table 2. Final models used to predict average monthly trihalomethane levels in the study population

Chemical/ cohort	model	Transformation of dependent variable	Variables	R2
Total THM				
Asturias	GAM	Squared root	s(month), water source, municipality	0.448
Gipuzkoa	GAM	-	s(month), year, sample site	0.685
Granada	Linear	-	Municipality	0.905
Sabadell	GAM	Logarithm	s(coordinates) [a], s(month)	0.506
Valencia	GAM	Logarithm	s(month), population center	0.809
CHCl3				
Asturias	GAM	Square root	s(month), water source, municipality	0.530
Gipuzkoa	GAM	Square root	s(month), year, sample site	0.693
Granada	Linear	Logarithm	Municipality	0.900
Sabadell	GAM	Squared root	s(coordinates) [a], Month, year	0.391
Valencia	GAM	Logarithm	s(month), population center	0.838
CHCl2Br				
Asturias	GAM	Squared root	s(month), water source, municipality	0.244
Gipuzkoa	GAM	-	s(month), sample.site	0.721
Granada	Linear	-	Municipality	0.883
Sabadell	GAM	Squared root	s(coordinates) [a], s(month)	0.295
Valencia	GAM	Logarithm	s(month), population center	0.817
CHClBr2				
Asturias	GAM	Squared root	s(month), year, municipality	0.331
Gipuzkoa	Linear	-	Sample site	0.670
Granada	Linear	Logarithm	Municipality	0.899
Sabadell	GAM	Squared root	s(coordinates) [a], Month, year	0.563
Valencia	GAM	Logarithm	s(month), population center	0.792
CHBr3				
Asturias	GAM	Squared root	s(month), year, municipality	0.469
Gipuzkoa	GAM	Logarithm	s(month), year, sample site	0.551
Granada	Linear	Logarithm	Municipality	0.750
Sabadell	GAM	$()^{\wedge 3/4}$	s(coordinates) [a], Month	0.468
Valencia	GAM	Logarithm	s(month), population center	0.786

a. Coordinates of the residence from all study subjects were available only in Sabadell.

s(variable) = smoothed function

GAM. Generalized additive model

Supplemental Material, Table 3. Uptake factors from the literature and values used in this study.

Compound	Ingestion		Showering		Bathing		Swimming	
	Uptake factors based on the literature [a]							
	Uptake factor (μg/μg/l)	References	Uptake factor (μg/min/μg/l)	References	Uptake factor (μg/min/μg/l)	References	Uptake factor (μg/min/μg/l)	
CHCl3	0.004901961	Backer et al. 2000	0.001536261	Backer et al. 2000<br>Lynberg et al. 2001	0.001320755	Backer et al. 2000	0.002541407	Aggazzotti 1995
CHCl2Br	0.001086957	Backer et al. 2000	0.001315092	Backer et al. 2000<br>Lynberg et al. 2001	0.001189711	Backer et al. 2000	-	
CHClBr2	0.00115	Backer et al. 2000	0.001389034	Backer et al. 2000<br>Lynberg et al. 2001	0.001401709	Backer et al. 2000	-	
CHBr3	-		0.000437931	Lynberg et al. 2001	-		-	
	Uptake factors used in the present analysis [b]							
CHCl3	0.00490196		0.00153626		0.00132075		0.00254141	
Brominated THM	0.00111848		0.00135206		0.00129571		0.00223672	

a. Values obtained from studies reporting blood THM levels after drinking, showering, bathing and swimming in a pool. For each study, water use and THM chemical, blood concentration was calculated for a given concentration in the water and liters of water ingested or duration of showering/bathing/swimming. Information was obtained either directly from the published paper or personal communication with the authors. When more than one factor was obtained for a given THM and water use, an average was calculated.

b. Brominated THM uptake factors are the average of bromodichloromethane and dibromochloromethane (and also bromoform for showering). Uptake factor for brominated THM in swimming pools was based on expert knowledge.

Supplemental Material, Figure 1. Boxplots of the total trihalomethane levels in indoor and outdoor swimming pools sampled in the study areas.

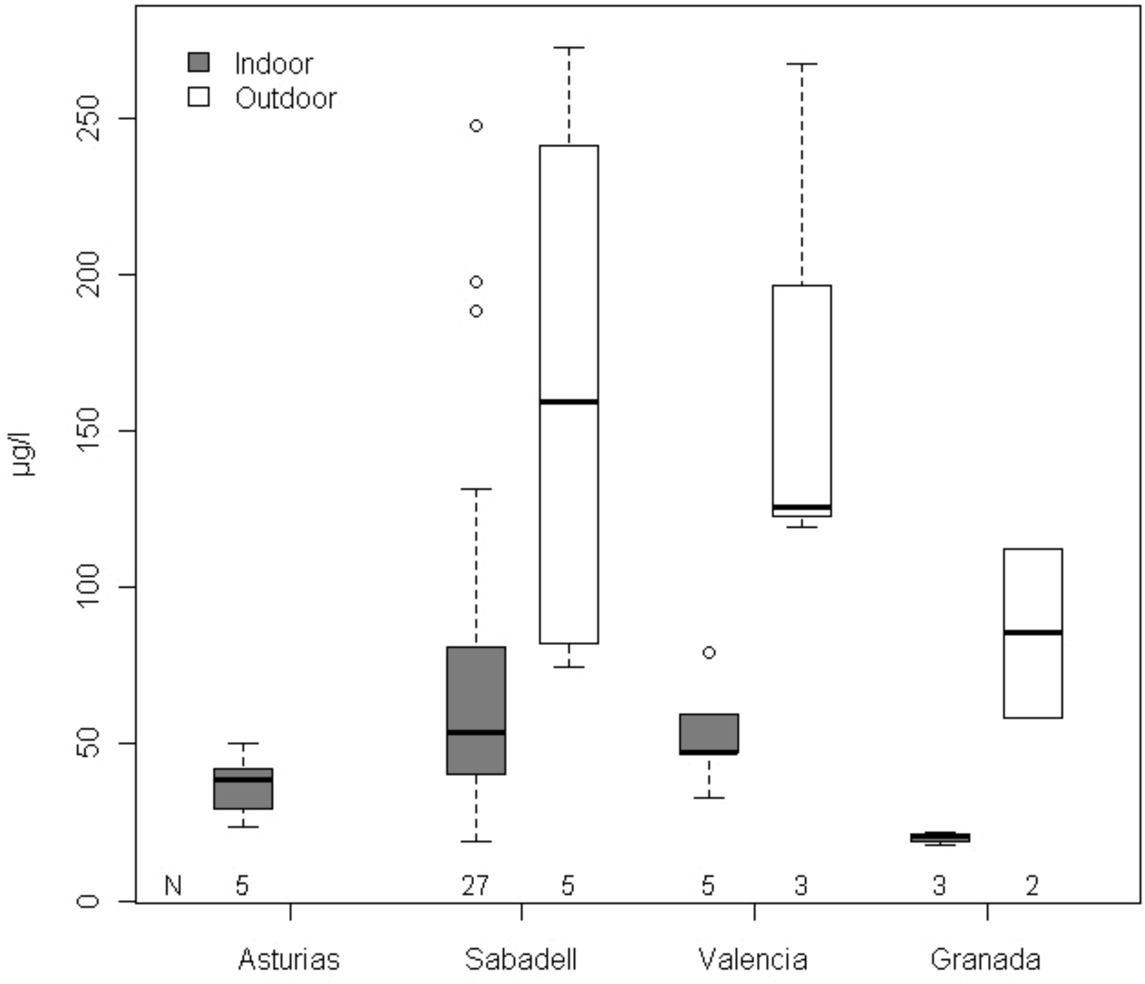

Supplemental Material, Figure 2. Total trihalomethane levels in the study areas. Average levels are presented for each district in Sabadell, and each municipality in Asturias, Gipuzkoa, Valencia and Granada. The grey circles represent the average level and the black circles inside represent the standard error. The white circle at the top right indicates the scale of concentrations in each area. The number of samples (N) in each cohort are indicated

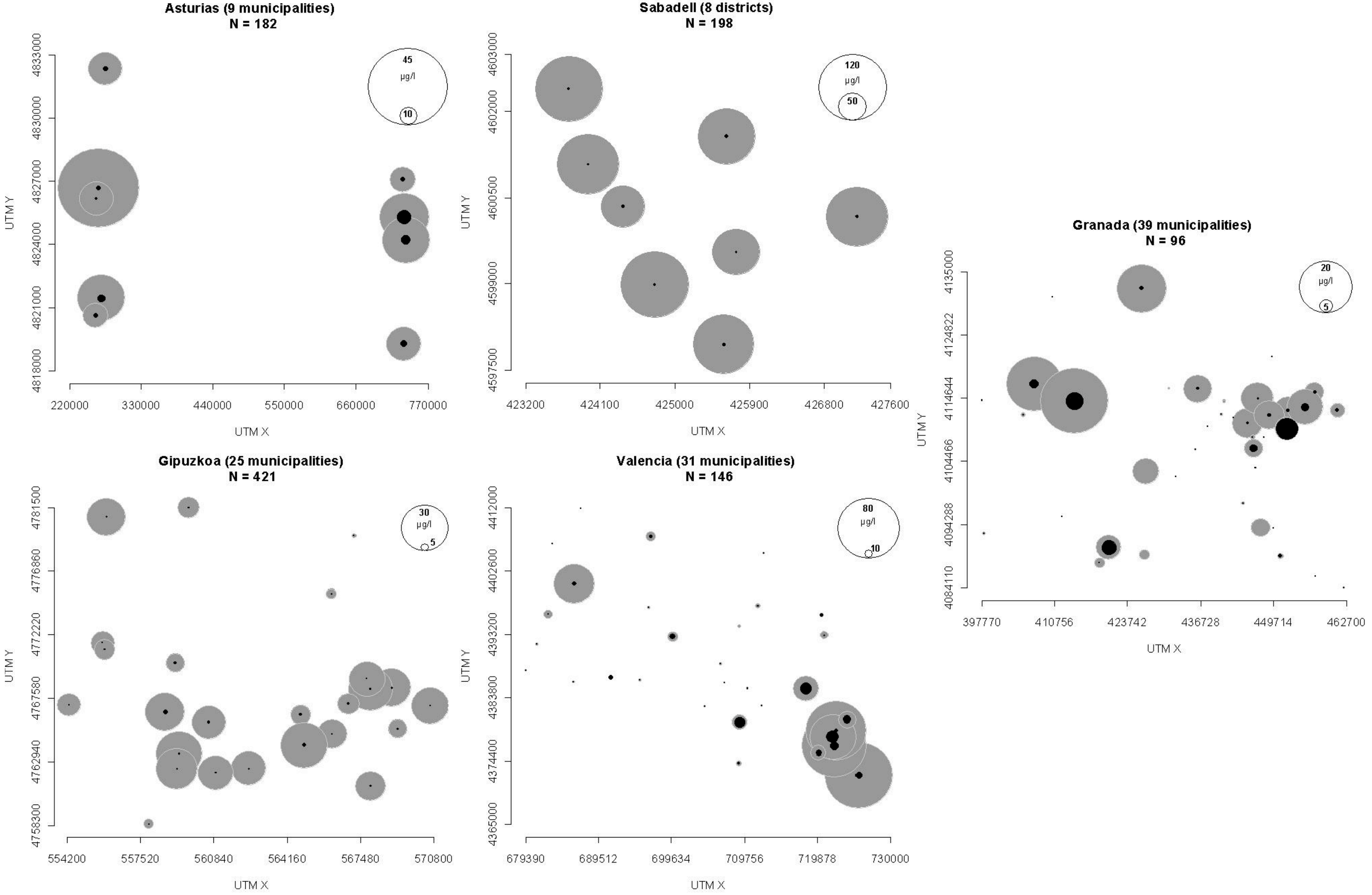

References

Aggazzotti G, Fantuzzi G, Righi E, Predieri G. 1995. Environmental and biological monitoring of chloroform in indoor swimming pools. J Chromatogr A 710:181-190.

Backer LC, Ashley DL, Bonin MA, Cardinali FL, Kieszak SM, Wooten JV. 2000. Household exposures to drinking water disinfection by-products: whole blood trihalomethane levels. J Expo Anal Environ Epidemiol 10:321-326.

Font-Ribera L, Esplugues A, Ballester F, Martinez B, Tardon A, Freire C, et al. 2010. Trihalomethanes in swimming pool water in four areas in Spain 1 participating in the INMA project. Gac Sanit 24:483-486.

Lynberg M, Nuckols JR, Langlois P, Ashley D, Singer P, Mendola P et al. 2001. Assessing exposure to disinfection by-products in women of reproductive age living in Corpus Christi, Texas, and Cobb County, Georgia: descriptive results and methods. Environ Health Perspect 109:597-604.

Santa Marina L, Ibarluzea J, Basterrechea M, Goni F, Ulibarrena E, Artieda J et al. 2009. Indoor air and bathing water pollution in indoor swimming pools in Guipuzcoa (Spain). Gac Sanit 23:115-120.
